# The role of energy in health facilities: A conceptual framework and complementary data assessment in Malawi

**DOI:** 10.1371/journal.pone.0200261

**Published:** 2018-07-20

**Authors:** Laura Suhlrie, Jamie Bartram, Jacob Burns, Lauren Joca, John Tomaro, Eva Rehfuess

**Affiliations:** 1 Institute for Medical Information Processing, Biometry and Epidemiology, Pettenkofer School of Public Health, Ludwig Maximilians University Munich, Munich, Bavaria, Germany; 2 The Water Institute, Gillings School of Global Public Health, University of North Carolina at Chapel Hill, Chapel Hill, North Carolina, United States of America; Pan-Atlantic University, NIGERIA

## Abstract

**Background:**

Modern energy enables health service delivery. Access to electricity is, however, unreliable in many health facilities in developing countries. Little research has explored the relationships between energy and service delivery.

**Methods:**

Based on extensive literature searches and iterative discussions within the research team, we first develop a conceptual framework of the role of energy in health facilities. We then use this framework to explore how characteristics of electricity supply affect distinct energy uses in health facilities (e.g. lighting), and how functional or non-functional lighting affects the provision of night-time care services in Malawi. To do so we apply descriptive statistics and conduct logistic and multinomial regressions using data from the Service Provision Assessment (SPA) of the Demographic and Health Surveys (DHS) for all health facilities in Malawi in 2013/2014.

**Results:**

The conceptual framework depicts the pathways from different energy types and their characteristics, through to distinct energy uses in health facilities (e.g. medical devices) and health-relevant service outputs (e.g. safe medical equipment). These outputs can improve outcomes for patients (e.g. infection control), facilities (e.g. efficiency) and staff (e.g. working conditions) at facilities level and, ultimately, contribute to better population health outcomes. Our exploratory analysis suggests that energy uses were less likely to be functional in facilities with lower-quality electricity supply. Descriptive statistics revealed a critical lack of functional lighting in facilities offering child delivery and night-time care; surprisingly, the provision of night-time care was not associated with whether facilities had functional lighting. Overall, the DHS SPA dataset is not well-suited for assessing the relationships depicted within the framework.

**Conclusion:**

The framework conceptualizes the role of energy in health facilities in a comprehensive manner. Over time, it should be empirically validated through a combination of different research approaches, including tracking of indicators, detailed energy audits, qualitative and intervention studies.

## Introduction

Access to modern energy for lighting, cooking, heating and powering appliances at the level of households as well as institutions is crucial to socio-economic development. “Modern energy” comprises electricity and less polluting, safer forms of thermal energy (e.g. improved stoves) [1–2]. Although significant progress has been made towards ensuring access to modern energy world-wide, sub-Saharan Africa has the highest population shares without electricity and relying on polluting solid fuels, especially in rural areas [3–5]. These conditions, in particular a lack of electricity, affect health facilities and the services they provide: In 13 health facility surveys among 11 sub-Saharan African countries, 74% of health facilities reported having access to electricity; in eight of these countries, only 28% of health facilities reported having continuous access to electricity [6].

To address this deficit, the United Nations (UN) launched the Sustainable Energy For All (SEforAll) initiative in 2011 and formulated the energy-specific goal 7 of the Sustainable Development Goals (SDGs), adopted in 2015. Both efforts aim to bring together multiple sectors to: achieve universal access to affordable and reliable modern energy services; increase the share of renewable energy technologies; and improve energy efficiency by 2030 [2,7]. Within the health sector, these goals are fostered by the High Impact Opportunity (HIO) on Energy for Women´s and Children´s Health, launched as part of SEforALL, which aims to improve energy access in health facilities to improve child and maternal health service delivery [8].

While much research is dedicated to assessing the health impacts of polluting fuels and to promoting effective household solutions, few investigations explore the roles of energy in health facilities. The World Health Organization (WHO) emphasizes that modern energy uses are crucial for providing basic health services and for ensuring safe working conditions for health personnel [9]. A few studies point to the specific roles of energy for lighting, vaccine storage, sterilization and communications in health facilities [10–12]. A report published by WHO and the World Bank offers a comprehensive overview of the status and trends of commonly used energy types in health facilities in resource-constrained settings [13]. However, there is no comprehensive assessment of the impact of energy access and type in health facilities on specific energy uses, and consequently on health service delivery.

In this paper, we first develop a conceptual framework that describes the pathways from different energy types and their characteristics, to distinct energy uses in health facilities (e.g. medical devices, technologies for disinfection and sterilization) and health-relevant service outputs (e.g. advanced diagnostics and treatment, safe medical equipment), to outcomes for patients (e.g. infection control), facilities (e.g. efficiency) and staff (e.g. working conditions) at facilities level and, finally, population health outcomes (objective 1). We then use this framework to explore how the characteristics of the electricity supply affect distinct energy uses in health facilities (objective 2a), and, in a further step, how functional or non-functional lighting affects the provision of night-time care services offered in Malawi (objective 2b).

## Methods

### Conceptual framework (objective 1)

**Fig 1:**
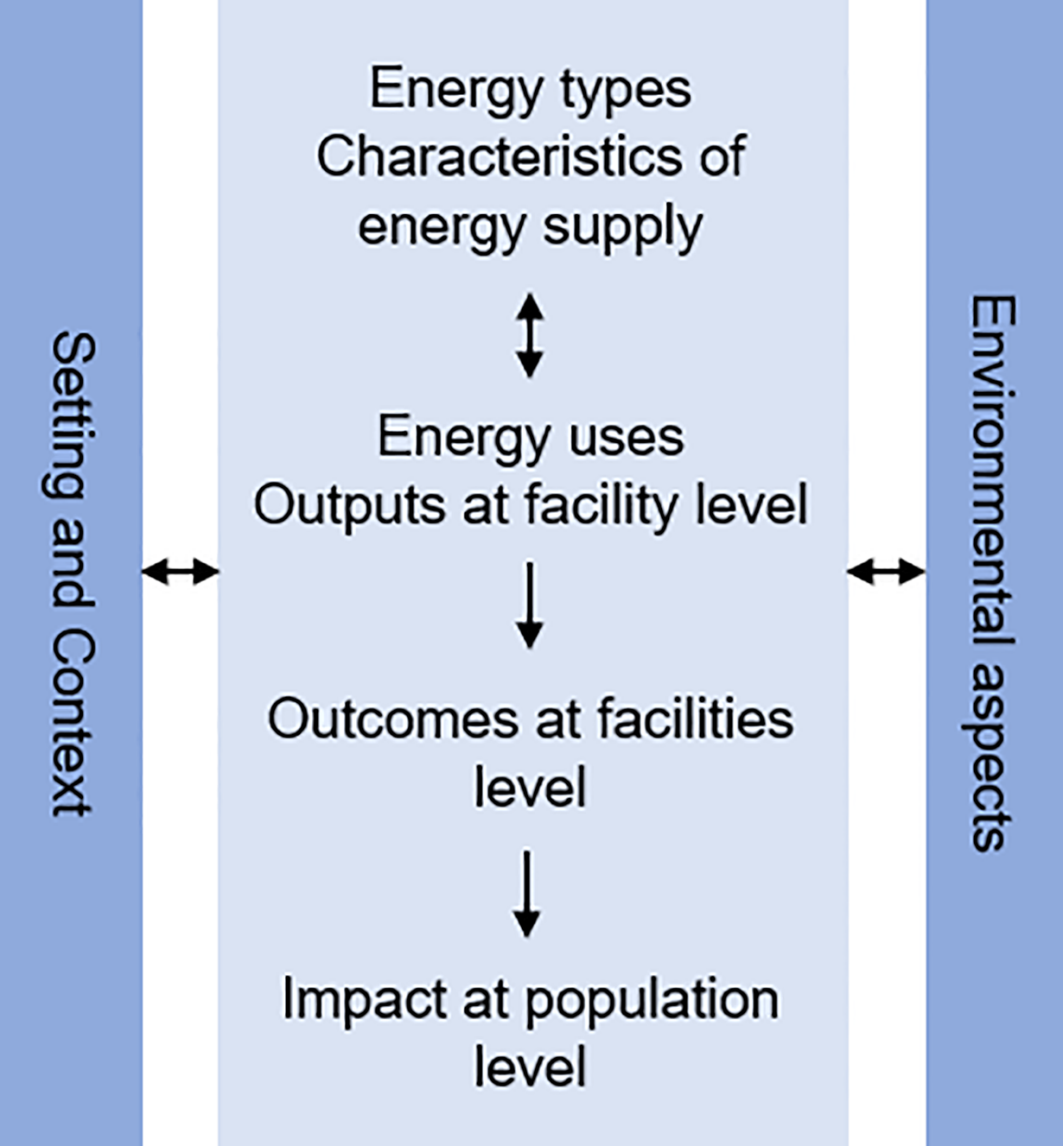
Structure of the conceptual framework of the role of energy in health facilities.

An extensive literature search was conducted to retrieve studies examining the role of energy in health facilities. This search consisted of a mix of structured searches of electronic databases, as well as snowball searches based on key publications identified. When first defining the scope of this project, we identified two reports that were especially informative [6,13]: The first made use of the SPA to outline the critical energy situation in health facilities in sub-Saharan Africa, and the second represents the first comprehensive assessment of energy in health facilities. We used these reports to develop the keywords for the structured searches, which included “health facilit*”, “hospital”, “clinic*”, “health service delivery”, “electricity”, “energy access”, “developing countr*”. Using combinations of these keywords, we searched PubMed, EBSCO Host Database Academic and Google Scholar. All study types providing information on the role of energy in health facilities were considered relevant. Informed by all relevant studies identified through these searches and literature on logic models and framework-based approaches [14–16], we developed a structure of the conceptual framework comprising the following domains at four levels: energy types and characteristics of supply; energy uses and outputs at facility level; outcomes at facilities level; and impact on population level. These domains interact with additional domains, i.e. facility setting (physical location), context (overarching macro- and meso-level aspects) and environment as well as environmental health aspects (Fig 1).

This structure was populated with categories (boxes) and sub-categories (bullet points within boxes) by LS in discussion with all members of the research team in an iterative process. Where we could not retrieve relevant information from the literature, in particular with respect to how energy uses affect outputs, we discussed and agreed upon assumptions (presented in italics).

### Data and variables for exploratory analysis (objective 2)

#### Data source

Data for the exploratory analysis were drawn from the Service Provision Assessment (SPA) surveys, which are conducted by the Demographic and Health Survey (DHS) program of the United States Agency for International Development (USAID); these data are in the public domain [17]. The SPA surveys comprise four components: an inventory questionnaire, healthcare provider interviews, observations of consultations and patient interviews. The last two are applied in the fields of antenatal care, family planning and treatment of sick children. The provider interviews collect details on the health professionals, such as educational background and services offered. The inventory questionnaire collects information about the infrastructure, resources and systems of the facility to ascertain USAID and WHO service readiness indicators [18].

We used the most recent data from the inventory questionnaire for Malawi from 2013/2014, which was set up as a complete survey of all health facilities [19]. Out of 1066, 977 health facilities filled in the questionnaire. Reasons for non-response were: that facilities refused to be assessed (3%) or had closed down (2%), that no-one was available to respond to the survey (1%) and that facilities were inaccessible for various reasons (2%) (DHS, personal communication). Five further facilities were excluded because information about the energy source and its continuity was missing. Data from the remaining 972 facilities were weighted to account for differentials regarding different facility types caused by this non-response.

For the 972 included observations, we retrieved variables that could serve as measures or proxies for the categories and sub-categories defined in the top three levels of the conceptual framework, i.e. energy types and characteristics of supply, energy uses and outputs at facility level and outcomes at facility level; the fourth level, the impact on population level, could not be populated using data from the SPA inventory survey.

#### Energy types and characteristics of supply

The SPA inventory survey provides information on electricity but not on thermal energy. Based on the available data, we characterized the electricity source and its continuity as: uninterrupted grid with back-up; interrupted grid with back-up; uninterrupted grid without back-up; interrupted grid without back-up; off-grid electricity; and no electricity.

Fuel-based generators may serve as back-up sources or as the main electricity source in off-grid settings. Solar systems may also constitute the main off-grid electricity source, either alone or combined with fuel-based generators. A proxy for the continuity of electricity supply in SPA surveys is only provided for grid-connected facilities: a continuous energy supply (i.e. “uninterrupted grid”) is defined as having had no interruptions of more than two hours during the opening hours of the facility in the last 7 days.

#### Energy uses

Thirteen variables related to seven of the total of eight *Energy uses* in the conceptual framework were identified. These comprise *lighting in outpatient area* and *lighting in delivery area (for lighting); light microscope*, *hematology analyzer and newborn incubator (for medical devices); water source* and *sanitation in outpatient area (for water pumps and (wastewater) treatment); communication technologies*, *computer*, *computer and internet combined (for information and communication technologies* (ICT)*); vaccine refrigeration (for refrigeration); technologies for disinfection and sterilization (for technologies for disinfection*, *sterilization and healthcare waste treatment* (HCWT)*);* and *ventilation in medication storage room (for heating*, *ventilation and air conditioning* (HVAC)*)*. The variables identified for *lighting*, *medical devices*, *ICT*, *technologies for disinfection*, *sterilization and HCTW* assessed first whether the specific energy uses were available and then whether these were functional. As internet connectivity is a prerequisite to using a computer as an information technology, the outcome *computer and internet combined* assessed whether the facility had both a functional computer and internet available. The dataset did not include comprehensive information on ventilation and air conditioning; we therefore included the ventilation status of the medication storage room as a proxy. The three medical devices *hematology analyzer*, *newborn incubator* and *light microscope* were chosen based on a list of priority medical devices defined by WHO and UNICEF [9].

#### Outputs and outcomes at facility level

Based on the conceptual framework, we identified three variables for health service delivery potentially related to the availability of electricity and specific energy uses from the data: *night-time care services offered*, *child vaccination services offered*, and the *total number of outpatient visits in the last complete calendar month (both adult and children)*. However, in Malawi, child vaccination services are predominantly supported through campaigns and therefore not strictly linked with services of a health facility. We excluded the variable outpatient visits due to poor data quality: 113 (11.63%) facilities of the 972 facilities reported that they did not know the number of outpatient visits in the preceding month; it is unlikely that these excluded facilities are randomly distributed. Our exploratory analysis was therefore concerned with *night-time-care offered*.

#### Facility setting and context

Given expected heterogeneity in energy types as well as energy uses and outputs, across different health facilities and locations in Malawi, the variables *facility level*, *managing authority*, *region* and *urban-rural location* were included in all statistical analyses. The Malawian health system recognizes nine facility types; these were categorized into three levels of care as defined by WHO and the World Bank: Community level facilities include maternity facilities, dispensaries, clinics and health posts; first-level facilities comprise rural/community hospitals, other hospitals and health centres; and referral-level facilities consist of central and district hospitals [20–22].

#### Quantitative data analysis: Electricity characteristics and distinct energy uses (objective 2a)

Logistic regression models were used to examine the associations between the electricity source and the functionality of energy uses. General energy uses (e.g. water pumps) were assessed for all facilities. Service-specific energy uses (e.g. newborn incubator within facilities offering child delivery services) were only analyzed for facilities offering these services.

The following model was applied for each of the thirteen identified energy uses:

$${\mathrm{logit}(y}_{j})=\beta_{0}+\beta_{1}{\mathrm{energy}\;\mathrm{type}}_{j}+\beta_{2}{\mathrm{facility}\;\mathrm{level}}_{j}+\beta_{3}\mathrm{managing}\;{\mathrm{authority}}_{j}+\beta_{4}{\mathrm{region}}_{j}+\beta_{5}\mathrm{urban}/\mathrm{rural}+\varepsilon_{j}$$

Where, for a given facility, j, the outcome y listed takes the value of 0 or 1 depending on whether the energy use is functional or not. β_1_ represents the coefficient of the six categories regarding the characteristics of electricity supply, β_2,_ β_3,_ and β_4_ represent the coefficients of the three facility levels, six managing authorities (relating to *facility type* in the conceptual framework) and three administrative regions respectively, and β_5_ is a dummy-variable for the urban-rural location (relating to *infrastructure* in the conceptual framework).

Model diagnostics included the likelihood ratio test and the Nagelkerke test for assessing the explanatory power of the model as well as a cross validation using bootstrapping samples with the leave-one-out method to ascertain the accuracy of the model. In addition, where applicable, a sensitivity analysis on the availability (rather than functionality) of the energy uses was conducted (S1 Appendix).

#### Quantitative data analysis: Lighting in delivery area and night-time care (objective 2b)

Night-time care is of particular importance for child delivery services because of the time-sensitive need for health services around and during childbirth [23]. We assessed whether lighting in the delivery area is associated with night-time care offered in facilities providing child delivery services.

The following multinomial logistic model was applied:

$${f(k}_{j})=\beta_{0}+\beta_{1}\mathrm{lighting}\;\mathrm{delivery}\;{\mathrm{area}}_{j}+\beta_{2}{\mathrm{facility}\;\mathrm{level}}_{j}+\beta_{3}\mathrm{managing}\;{\mathrm{authority}}_{j}+\beta_{4}{\mathrm{region}}_{j}+\beta_{5}\mathrm{urban}/\mathrm{rural}+\varepsilon_{j}$$

where for a given facility, j, the outcome k listed takes the value of 0 or 1 depending on whether the facility offers the following services: no inpatient/overnight services, overnight observation services or inpatient services. β1 represents the coefficient of the independent variable of whether lighting in the delivery area is functional or not, and β2 to β5 display the coefficients of the above described variables.

The same diagnostic tests as for objective 2a were applied, including a sensitivity analysis for the availability (rather than functionality) of lighting in the delivery area.

All statistical analyses were carried out using R Studio (Version 1.0.143).

## Results

### Conceptual framework (objective 1)

**Fig 2:**
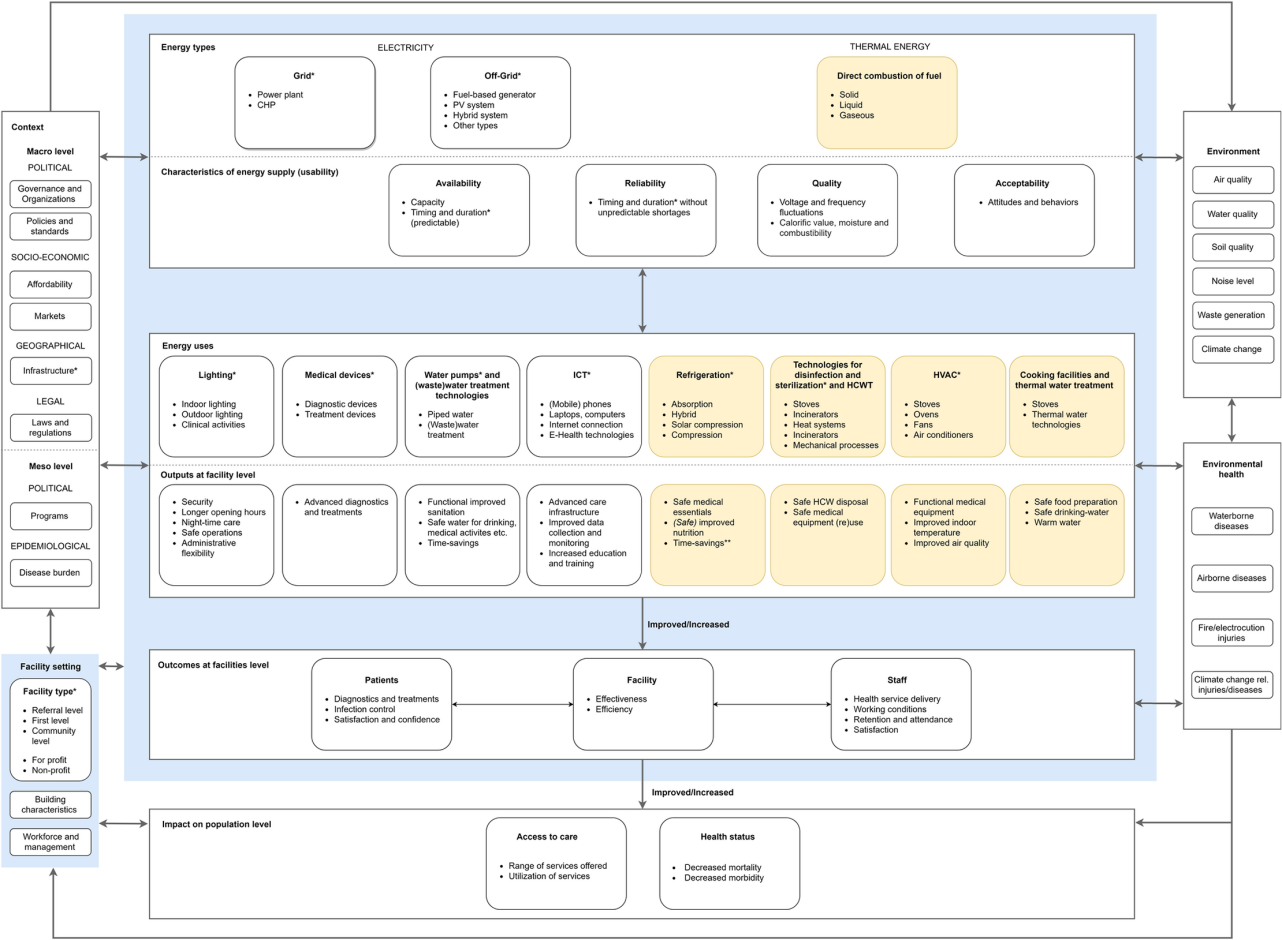
Conceptual framework of the role of energy in health facilities. Blue shading: Processes taking place within the health facility Yellow shading: Energy uses not necessarily depending on electricity, except for uses in grey font color *: Included in the quantitative analysis **: Assumptions made by the authors. Abbreviations: CHP: Combined Heating Power ICT: Information and Communication Technologies PV: Photovoltaic HCWT: Healthcare Waste Treatment HVAC: Heating, Ventilation and Air Conditioning.

Fig 2 presents our comprehensive conceptual framework of the role of energy in health facilities. Three main energy types: grid- and off-grid electricity as well as thermal energy can be used as main or back-up sources. Their characteristics include availability (capacity and predictable timing and duration), reliability (timing and duration without unpredictable shortages), quality (voltage quality and combustibility) and acceptability (attitudes and behavior). These characteristics affect the energy uses *lighting*, *medical devices*, *water pumps and (waste)water treatment technologies*, *information and communication technologies*, *refrigeration*, *technologies for sterilization*, *disinfection and healthcare waste treatment*, *heating*, *ventilation and air conditioning*, *cooking facilities and thermal water treatment technologies*. These energy uses in turn enable specific health-relevant service outputs at facility level (e.g. extended opening hours through lighting). In turn, these outputs improve the infrastructure and functionality of the health facility and contribute to the facility-level outcomes of meeting the needs of patients and staff. Ultimately, at population level, energy in health facilities contributes to both improved access to health services and a better health status of the population. External factors, such as the facility setting, the context (e.g. political, socio-economic) and environmental as well as environmental health aspects influence, and are influenced by, the availability and energy use in health facilities. The various interactions depicted in the conceptual framework operate across all levels and domains.

### Electricity characteristics and their impacts on health facilities in Malawi

#### Descriptive results

Table 1 displays the distribution of all variables. While 69% of all health facilities in Malawi were connected to the grid, 9% had no electricity. Among all grid-connected facilities, 29% had a back-up source, which was functional in almost all cases (data on functionality of back-up source not shown). Only 38% of the facilities with an off-grid main source indicated that the generator or combined system was functional on the day of data collection.

**Table 1:** Descriptive statistics of all variables included in the data analysis.

		n	%
**Energy types and**	C1: Uninterrupted grid with back-up	72	7.41
**characteristics of**	C2: Interrupted grid with back-up	152	15.64
**energy supply**^**1**^	C3: Uninterrupted grid without back-up	122	12.55
	C4: Interrupted grid without back-up	325	33.44
	C5: Off-grid electricity source	218	22.43
	C6: No electricity source	83	8.54
	Total	972	100.00
**Facility setting and**	***Facility level***^***2***^		
**context**	Community level	402	41.36
	First level	543	55.86
	Referral level	27	2.78
	Total	972	100.00
	***Managing authority***		
	Government/public	472	48.56
	Christian Health Association of Malawi (CHAM)	154	15.84
	Private for profit	212	21.81
	Mission/Faith-based (other than CHAM)	7	0.82
	Non-governmental	58	5.97
	Company	68	7.00
	Total	972	100.00
	***Region***		
	North	167	17.18
	Central	361	37.14
	South	444	45.68
	Total	972	100.00
	***Urban-rural location***		
	Urban	300	30.86
	Rural	672	69.14
	Total	972	972
**Energy uses**	***Lighting in outpatient area***^***3***^		
	Functional	362	37.24
	Not functional	588	60.49
	Question not applicable (no outpatient serv. offered)	22	2.26
	Total	972	100.00
	***Lighting in delivery area***^***4***^		
	Functional	181	18.62
	Not functional	355	36.52
	Question not applicable (no delivery serv. offered)	436	44.86
	Total	972	100.00
	***Water source***		
	Piped water	682	70.16
	Other improved water source^5^	260	26.75
	Unimproved water source^6^	18	1.85
	No water source	9	0.93
	Other^7^	3	0.31
	Total	972	100
	***Sanitation in outpatient area***		
	Flush to piped sewer system or septic tank	258	26.54
	Other improved sanitation^8^	592	60.91
	Unimproved sanitation^9^	78	8.02
	No functioning sanitation facility	22	2.26
	Question not applicable (no outpatient serv. offered)	22	2.26
	Total	972	100.00
	***Ventilation in medication storage room***		
	Well ventilated	843	86.73
	Not well ventilated	116	11.93
	Question not applicable (no medication stored)	13	1.34
	Total	972	100.00
	***Computer***		
	Functional	300	30.86
	Not functional	672	69.14
	Total	972	100.00
	***Computer and internet***^***10***^ ***combined***		
	Functional computer and internet available	243	25.00
	No funct. computer and/or no internet available	729	75.00
	Total	972	100.00
	***Communication technologies***^***11***^		
	Functional	804	82.72
	Not functional	168	17.28
	Total	972	100.00
	***Light microscope***		
	Functional	200	20.58
	Not functional	600	61.73
	Question not applicable (no diagnostic serv. offered)	172	17.70
	Total	972	100.00
	***Hematology analyzer***		
	Functional	177	18.21
	Not functional	90	9.26
	Question not applicable (no hemoglobin test. offered)	705	72.53
	Total	972	100.00
	***Newborn incubator***		
	Functional	37	3.81
	Not functional	499	51.34
	Question not applicable (no delivery serv. offered)	436	44.86
	Total	972	100.00
	***Vaccine refrigeration***		
	Appropriate temperature (2–8°C)	457	47.02
	Inappropriate temperature	125	12.86
	Thermometer not functional	33	3.40
	Refrigeration not available	10	1.03
	Question not applicable (no child vaccination serv. offered)	347	35.70
	Total	972	100.00
	***Technologies for sterilization and disinfection***^***12***^		
	Functional	375	38.58
	Not functional	418	43.00
	Question not applicable (no sterilization and disinfection only within the facility)	179	18.42
	Total	972	100.00
**Outputs at facility**	***Night-time care***		
**Level**	No overnight observation/inpatient services	50	5.14
	Overnight observation	257	26.44
	Inpatient services	202	20.78
	Question not applicable (no delivery serv. and no outpatient serv. offered)	463	47.63
	Total	972	100.00

^1^C1: Uninterrupted grid with back-up; C2: Interrupted grid with back-up; C3: Uninterrupted grid without back-up; C4: Interrupted grid without back-up; C5: Off-grid electricity source; C6: No electricity source

Interrupted grid: Interruption of more than two hours at a time in the last seven days during service hours

Back-up sources of grid-connected facilities: Solar power, battery- or fuel-based generator

Off-grid electricity sources: Solar power, battery- or fuel-based generators or combined electricity sources

^2^ Community level: Maternity facilities, dispensaries, clinics, health posts

First level: Rural/Community hospitals, other hospitals, health centres

Referral level: Central hospitals, district hospitals

^3^ Incl. flashlights

^4^ Incl. flashlights

^5^ Other improved water sources: public tap/standpipe, protected well, tubewell/borehole, protected spring, rain water

^6^ Unimproved water sources: unprotected well, river/lake/pond, cart/small tank/drum, tanker trunk

^7^ Recoded as missing in quantitative analysis

^8^ Other improved sanitation: flush to pit latrine, ventilated improved pit latrine, pit latrine with slab

^9^ Unimproved sanitation: Flush to somewhere else, pit latrine without slab/open pit

^10^ n = 50 internet less than 2 hours available, n = 344 internet more than two hours available (during operation hours)

^11^ Landline, cellphone or shortwave radio

^12^ Includes facilities which use only electrical devices for reuse and facilities which use both, non-electrical and electrical technologies for reuse.

Electrical processing technologies: Electric autoclave, electric dry-heat sterilizer, electric boiler or steamer

Non-electrical processing technologies: Non-electric autoclave, non-electric pot with cover for boiling/steam, stove or cooker

Table 2 shows wide variation in the electricity type and continuity between levels of health facilities: Many community-level (49%) and first-level facilities (64%) are connected to interrupted grid electricity without a back-up source; 11% and 7% of community-level and first-level facilities respectively do not have any access to electricity. In contrast, all referral-level facilities are grid-connected with a back-up source.

**Table 2:** Electricity sources and continuity by covariate (in percent).

		*C1*	*C2*	*C3*	*C4*	*C5*	*C6*
**Facility**	*Community level*	8.53	13.38	18.00	39.92	9.09	11.07
**level**	*First level*	4.67	9.86	14.69	30.34	33.29	7.17
	*Referral level*	46.43	53.57	0	0	0	0
**Managing**	*Government/public*	4.15	5.95	15.61	28.04	33.45	12.79
**authority**	*CHAM*^*1*^	8.87	20.91	10.71	26.60	27.87	5.04
	*Private for profit*	5.72	14.92	17.39	50.44	4.81	6.73
	*Mission/Faith-based*^*2*^	43.64	27.30	0	14.53	0	14.53
	*Non-governmental*	10.43	19.14	17.83	45.59	7.01	0
	*Company*	25.77	24.52	21.44	25.23	3.04	0
**Region**	*North*	2.94	10.60	9.00	40.16	28.38	8.91
	*Central*	8.57	11.61	16.01	25.86	29.47	8.43
	*South*	8.15	13.97	17.77	37.09	14.44	8.59
**Urban-rural**	*Urban*	16.71	22.04	18.42	40.12	1.68	1.03
**location**	*Rural*	3.27	8.28	14.42	30.49	31.58	11.96

C1: Uninterrupted grid with back-up; C2: Interrupted grid with back-up; C3: Uninterrupted grid without back-up; C4: Interrupted grid without back-up; C5: Off-grid electricity source; C6: No electricity source

^1^ Christian Health Association of Malawi

^2^ Other than CHAM

Government facilities tend to be connected to off-grid systems, and those connected to the grid have fewer back-up sources compared to facilities operated by other managing authorities. Facilities in the Northern Region show less electricity access than facilities in the Central and Southern Regions, both in relation to being grid-connected with continuous supply and in relation to having a back-up source. As expected, electricity access and continuity are generally better in urban compared to rural areas.

#### Electricity characteristics and distinct energy uses (objective 2a)

Table 3 shows the results of the logistic regressions on the functionality of lighting in outpatient and delivery areas. In both areas, diverting from the reference category of uninterrupted grid supply with a back-up source, the remaining energy categories are associated with a decrease in the functionality of lighting with the strongest effects seen for off-grid electricity and no electricity source. Indeed, facilities with off-grid supply and facilities without electricity were approximately 80% and 90% less likely to have functional lighting in outpatient and delivery areas respectively, independent of facility level, managing authority, region and urban-rural location compared to the reference category (p<0.01).

**Table 3:** Lighting in outpatient and child delivery areas: Odds ratios and 95% confidence intervals of energy source and continuity.

Outcome variables	Lighting in outpatient area^1^		Lighting in child delivery area^2^	
	Functional (= 1) vs. Not functional		Functional (= 1) vs. Not functional	
	OR	(95% CI)	OR	(95% CI)
**Constant**	1.13	(0.48, 2.65)	2.04	(0.32, 12.81)
**C1**	ref		ref	
**C2**	0.65	(0.33, 1.26)	0.75	(0.29, 1.89)
**C3**	0.36***	(0.18, 0.71)	0.26***	(0.08, 0.71)
**C4**	0.29***	(0.15, 0.56)	0.38**	(0.14, 0.97)
**C5**	0.21***	(0.10, 0.43)	0.19***	(0.07, 0.50)
**C6**	0.10***	(0.04, 0.25)	0.04***	(0.01, 0.19)
**N**	950		536	
**LR test χ**^**2**^	165.56***		81.03***	
**Nagelkerke pseudo R**^**2**^	0.22		0.19	

C1: Uninterrupted grid with back-up; C2: Interrupted grid with back-up; C3: Uninterrupted grid without back-up; C4: Interrupted grid without back-up; C5: Off-grid electricity source; C6: No electricity source; OR: Odds ratio; CI: Confidence interval

LR: Likelihood ratio

*** p<0.01.

** p<0.05.

All regressions controlled for: Facility level, managing authority, region, urban/rural location

^1^ In facilities offering outpatient services

^2^ In facilities offering child delivery services

Table 4 presents the results of the logistic regressions on water source, sanitation facilities and ventilation in the medication storage room. Off-grid facilities were approximately 90% less likely to have piped water and a flush toilet compared to the reference category (p<0.01). Facilities connected to an interrupted grid without a back-up source were 90% less likely to have flush toilets (p<0.01). Similarly, off-grid connected facilities were 79% less likely to have a well-ventilated medication storage room compared to the reference category (p<0.05).

**Table 4:** Water source, sanitation facilities and ventilation in the medication storage room: odds ratios and 95% confidence intervals of energy source and continuity.

Outcome variables	Water source ^1^^,^^2^		Sanitation in outpatient area		Ventilation in medication storage room^3^	
	Piped into facility/on facility grounds (= 1) vs. other/no water source		Flushed toilets into sewer system/septic tank (= 1) vs. other/no sanitation facilities		Well ventilated (= 1) vs. not well ventilated	
	OR	(95% CI)	OR	(95% CI)	OR	(95% CI)
**Constant**	25.76***	(5.64, 188.94)	3.32**	(1.18, 9.73)	7.78***	(2.01, 41.13)
**C1**	ref		ref		ref	
**C2**	0.73	(0.10, 3.48)	0.58	(0.27, 1.21)	0.46	(0.10, 1.65)
**C3**	0.21	(0.03, 0.83)	0.33***	(0.14, 0.73)	0.45	(0.09, 1.67)
**C4**	0.23	(0.04, 0.86)	0.10***	(0.05, 0.22)	0.27	(0.06, 0.93)
**C5**	0.11***	(0.02, 0.41)	0.06***	(0.02, 0.15)	0.21**	(0.04, 0.75)
**C6**	0.05***	(0.01, 0.22)	0 cell^a^		0.25	(0.05, 0.98)
**N**	969		950		959	
**LR test χ**^**2**^	303.66***		463.57***		47.47***	
**Nagelkerke pseudo R^2^**	0.38		0.56		0.09	

C1: Uninterrupted grid with back-up; C2: Interrupted grid with back-up; C3: Uninterrupted grid without back-up; C4: Interrupted grid without back-up; C5: Off-grid electricity source; C6: No electricity source; OR: Odds ratio; CI: Confidence interval; LR: Likelihood ratio

*** p<0.01

** p<0.05

All regressions controlled for: Facility level, managing authority, region, urban/rural location

^1^ 3 observations deleted because water source was unclear

^2^ As electricity is only required in tall buildings to pipe against gravity, it can be assumed that a large proportion of examined facilities would not need electricity for piped water, thus the outcome might not fully reflect electricity access within the facility

^3^ In facilities which store medication and antibiotics

^a^ 0 cell in “not functional”

Table 5 shows the results of the logistic regressions on the functionality of computers alone and in combination with internet, as well as on the functionality of communication devices and specific medical devices. Grid-connected facilities without a back-up source and off-grid facilities were more than 90% less likely to have a functional computer (p<0.01), and even less likely to have a functional computer and internet, compared to the reference group (p<0.01). No significant association was found for the outcome on functional communication technologies. Grid-connected facilities without a back-up source were significantly less likely to have functional medical devices, given that the facility offered the specific service. For example, off-grid facilities were more than 90% less likely to have a functional newborn incubator or light microscope (p<0.01).

**Table 5:** Information and communication technologies and medical devices: Odds ratios and 95% confidence intervals of energy source and continuity.

Outcome variables	Computer		Computer and internet combined		Communication technologies		Light microscope^1^		Hematology analyzer^2^		Newborn incubator^3^	
	Functional (= 1) vs. Not functional		Functional (= 1) vs. Not functional^c^		Functional (= 1) vs. Not functional		Functional (= 1) vs. Not functional		Functional (= 1) vs. Not functional		Functional (= 1) vs. Not functional	
	OR	(95% CI)	OR	(95% CI)	OR	(95% CI)	OR	(95% CI)	OR	(95% CI)	OR	(95% CI)
**Constant**	5.26***	(1.84, 15.83)	1.38	(0.46, 4.15)	5.10	(1.82, 15.79)	1.18	(0.42, 3.35)	0.26	(0.04, 1.55)	0.05**	(0.00, 0.72)
**C1**	ref		ref		ref		ref		ref		ref	
**C2**	0.53	(0.22, 1.23)	0.45**	(0.20, 0.97)	1.71	(0.57, 5.05)	-0.63	(0.30, 1.30)	0.93	(0.41, 2.12)	1.48	(0.53, 4.30)
**C3**	0.12***	(0.05, 0.26)	0.12***	(0.05, 0.26)	0.88	(0.32, 2.20)	0.17***	(0.08, 0.37)	0.18***	(0.05, 0.59)	0.10**	(0.01, 0.57)
**C4**	0.08***	(0.03, 0.17)	0.09***	(0.04, 0.18)	0.82	(0.31, 1.96)	0.15***	(0.07, 0.30)	0.07***	(0.02, 0.23)	0.05***	(0.01, 0.25)
**C5**	0.02***	(0.01, 0.04)	0.01***	(0.00, 0.04)	0.75	(0.27, 1.87)	0.04***	(0.02, 0.01)	0 cell^b^		0.03***	(0.00, 0.23)
**C6**	0.01***	(0.00, 0.03)^a^	0 cell^b^		0.47	(0.16, 1.27)	0.04***	(0.01, 0.14)	0 cell^b^		0 cell^b^	
**n**	972		972		972		800		267		536	
**LR test χ^2^**	496.34***		490.63***		86.44***		201.76***		110.94***		117.63***	
**Nagelkerke pseudo R^2^**	0.56		0.59		0.14		0.33		0.49		0.49	

C1: Uninterrupted grid with back-up; C2: Interrupted grid with back-up; C3: Uninterrupted grid without back-up; C4: Interrupted grid without back-up; C5: Off-grid electricity source; C6: No electricity source; OR: Odds ratio; CI: Confidence interval; LR: Likelihood ratio

*** p<0.01

** p<0.05

All regressions controlled for: Facility level, managing authority, region, urban/rural location

^1^ In facilities offering laboratory services

^2^ In facilities offering hemoglobin testing services

^3^ In facilities offering child delivery services

^a^ Low cell count in “functional” (n = 3)

^b^ 0-cell in “not functional”

^c^ Computer functional and internet available (= 1) vs. no functional computer and/or no internet available.

The regression results of the multinomial regression on the temperature of the vaccine refrigerator in Table 6 show no association with the type of electricity supply and a low overall model fit (LR test p<0.1). Concerning the functionality of disinfection and sterilization technologies the results of the logistic regression suggest that grid-connected facilities without a back-up source (73% and 60% for those with/without grid interruptions respectively) and off-grid facilities (97%) are substantially less likely to have functional electric disinfection and sterilization devices compared to the reference category (P<0.01, apart from uninterrupted grid-connected facilities, where P<0.05).

**Table 6:** Vaccine refrigeration and technologies for sterilization and disinfection: Odds ratios and 95% confidence intervals of energy source and continuity.

Outcome variables	Vaccine refrigeration^1^								Disinfection and sterilization technologies^2^	
	Temperature appropriate between 2–8 degrees (= 1) vs.								Functional (= 1) vs. not functional^3^	
	Above 8 degrees		Below 2 degrees		Thermometer not functional		No vaccine refrigeration available			
	OR	(95% CI)	OR	(95% CI)	OR	(95% CI)	OR	(95% CI)	OR	(95% CI)
Constant	0.04***	(0.01, 0.30)	28.50***	(0.00, 0.32)	0.10**	(0.01, 0.88)	0.03	(0.00, 1.68)	2.25	(0.73, 7.36)
C1	ref		ref		ref		ref		ref	
C2	1.76	(0.34, 9.19)	1.51	(0.31, 7.39)	0.74	(0.11, 4.92)	0.61	(0.02, 17.91)	0.63	(0.24, 1.54)
C3	2.89	(0.57, 14.69)	0.60	(0.01, 3.64)	1.07	(0.18, 6.39)	0 cell		0.40**	(0.15, 0.96)
C4	2.42	(0.49, 11.90)	1.10	(0.24, 5.01)	0.58	(0.01, 3.34)	0 cell		0.27***	(0.11, 0.61)
C5	3.90	(0.77, 19.67)	1.48	(0.21, 7.37)	1.18	(0.20, 7.09)	3.06	(0.11, 83.83)	0.03***	(0.01, 0.08)
C6	3.22	(0.57, 18.34)	2.37	(0.43, 13.17)	2.01	(0.27, 15.96)	6.23	(0.22, 173.21)	0.02***	(0.00, 0.06) ^a^
n	625								793	
LR test χ^2^	78.66								350.68***	
Nagelkerke pseudo R^2^	0.14								0.48	

C1: Uninterrupted grid with back-up; C2: Interrupted grid with back-up; C3: Uninterrupted grid without back-up; C4: Interrupted grid without back-up; C5: Off-grid electricity source; C6: No electricity source; OR: Odds ratio; CI: Confidence interval; LR: Likelihood ratio

*** p<0.01

** p<0.05

All regressions controlled for: Facility level, managing authority, region, urban/rural location

^1^ In facilities offering child vaccination services

^2^ In facilities only processing within the facility

^3^ Includes facilities which use only electrical devices for reuse and facilities which use both, non-electrical and electrical technologies for reuse

^a^ Low cell count in “functional” (n = 2)

#### Lighting in delivery area and night-time care (objective 2b)

Table 7 shows that in more than 50% of all facilities offering child delivery services, lighting in the delivery area was not functional; this is even more pronounced among facilities offering overnight observation services with approximately 75% non-functional lighting.

**Table 7:** Lighting functionality in the delivery area in facilities offering night-time care and delivery services (in %).

		*N*	*Total*	*C1*	*C2*	*C3*	*C4*	*C5*	*C6*
**Inpatient**	*functional*	93	46.25	66.84	61.64	38.03	44.73	27.12	0.00
**Services**	*not functional*	109	53.75	33.16	38.36	61.97	55.27	72.88	100.00
**Overnight**	*functional*	65	25.18	49.92	20.94	26.01	33.02	21.38	9.81
**Observation**	*not functional*	192	74.82	50.08	79.06	73.99	66.98	78.62	90.19
**No inpatient/**	*functional*	16	31.42	100.00	49.28	16.33	41.24	24.98	0.00
**Overnight services**	*not functional*	34	68.58	0.00	50.72	83.67	58.76	75.02	100.00

C1: Uninterrupted grid with back-up; C2: Interrupted grid with back-up; C3: Uninterrupted grid without back-up; C4: Interrupted grid without back-up; C5: Off-grid electricity source; C6: No electricity source.

The results of the multinomial regression in Table 8 show that functional lighting in the delivery area was not significantly associated with night-time care services. The results indicate that the provided lighting infrastructure may play a minor role in relation to whether the service is provided or not. Furthermore, the fact that delivery and night-time care services are offered within a facility do not seem to lead to prioritization of functional lighting in these settings.

**Table 8:** Lighting in the delivery area and night-time care.

Outcome variables	No inpatient/overnight services offered (= 1) vs.			
	Overnight services offered		Inpatient services offered	
	OR	(95% CI)	OR	(95% CI)
**Constant**	42.82	(2.77, 661.16)	7.05	(0.64, 77.13)
**Functional lighting in delivery area (= 1)**	0.75	(0.37, 1.51)	1.45	(0.71, 2.96)
**N**	509			
**LR test χ**^**2**^	148.54***			
**Nagelkerke pseudo R**^**2**^	0.29			

OR: Odds ratio; CI: Confidence interval; LR: Likelihood ratio

*** p<0.01

** p<0.05

Regression controlled for: Facility level, managing authority, region, urban/rural classification

## Discussion

### Key findings

Our conceptual framework (Fig 2) on the role of energy in health facilities is, to our knowledge, the first attempt to capture systematically and comprehensively the pathways from energy types and their characteristics, to distinct energy uses in health facilities and health-relevant service outputs. This range of distinct energy uses (e.g. lighting, medical devices etc.) and outputs is critical for functional health facilities and for meeting the needs of patients and staff. In turn, this is expected to contribute to better access to health care (in terms of both the range of services offered and a more equitable utilization of these services by all members of the population) and to an improved health status of the population. The literature-based development of the framework showed that there is very little in-depth research of the interconnectedness of the various elements within the framework, in particular regarding the impact of energy on health service delivery outcomes.

The conceptual framework is intended to be universal and should thus be applicable to all facility types in developing countries as well as high-income countries. We note, however, that it is particularly suitable to examining the role of energy in resource-constrained areas.

Our assessment using the DHS SPA dataset for Malawi found that energy uses were less likely to be functional in facilities with lower-quality electricity supply (defined in terms of being on- or off-grid, continuity of supply and having a functional back-up source), independent of the facility level, managing authority and geographical location.

Descriptive statistics revealed a critical lack of lighting devices in health facilities offering child delivery- and night-time care. To our surprise, the provision of night-time care was not associated with whether facilities had functional lighting.

We could not identify an appropriate indicator to track the impact of energy on health service delivery outcomes due to the limited suitability of the DHS SPA dataset to assessing a broader set of relationships within the framework.

We believe that our findings can play an important role in (i) drawing attention to the importance of energy in health facilities, (ii) highlighting “blind spots” for which little or no information is available, (iii) suggesting and informing new primary quantitative or qualitative research to examine various areas of the framework, and, (iv) developing more informative indicators to track health-relevant contributions of energy availability, reliability and use in health facilities by taking into consideration the complex interplay of several influencing factors. Tracking outcomes on the health service delivery level will be crucial in assessing the impact of different strategies for improving access to modern energy and for providing policy-relevant information to decision-makers in resource-constrained settings.

### Strengths and limitations of conceptual framework

In developing the conceptual framework, particularly its structure, we drew from methodological literature on the development and application of logic models and other graphical frameworks [14–15]. Through extensive literature searches we aimed to identify all relevant research in this field. It is, however, possible that we missed relevant publications as well as unpublished research. Additionally, where evidence for relationships was lacking, we completed the framework using assumptions, following an iterative approach involving the whole research team. A different research team might have conceptualized the framework differently.

Previous frameworks have been limited to covering specific aspects of the role of energy in health facilities. WHO and the World Bank developed a results chain framework from energy investments towards the aim of reaching SDG 3 on “good health and well-being” and SDG 7 on “affordable and clean energy” [1,13]. A multi-tier framework developed by the SEforAll Initiative for assessing energy access on household level based on identified attributes of energy supply was adapted for the health setting by WHO and the World Bank [2,13]. Our framework goes beyond these existing frameworks by examining more comprehensively the role of energy within health facilities (e.g. energy types other than electricity, distinct uses) and by addressing the impacts of energy on health service delivery and health outcomes at multiple levels. In addition, it takes into account the role of setting and context and emphasizes relevant interconnections between all elements of the framework.

### Strengths and limitations of quantitative data analyses

Informed by the conceptual framework, associations between the source and continuity of electricity and different energy uses were quantitatively explored. These analyses controlled for relevant contextual factors and, where applicable and possible, were only conducted for those facilities where the specific energy-related services were offered. However, our analyses were exploratory in nature, therefore, findings should be interpreted with caution considering the limitations in the underlying dataset (see below). The strong associations observed imply that health service delivery might be constrained by a lack of energy and functional energy uses, but drawing causal inferences is challenging. For example, it is unclear to what extent the variables used to assess energy sources and continuity actually measure what they are intended to measure, and to what extent these simply reflect overall differences in levels of a range of resources in health facilities. Likewise, the observed associations only reflect a small proportion of the complex system depicted in the conceptual framework, and it can be assumed that other factors, such as qualified personnel, strongly influence the linkages between electricity supply and continuity and energy uses. Statistical limitations include p-value inflation due to the large number of regressions conducted and low cell counts or 0-cells in some categories of the energy type variable.

The SPA dataset provides information on the electricity source and its continuity, as well as a majority of the identified energy uses in our framework, although the data obtained from the SPA are characterized by limitations. Regarding electricity sources and their reliability, first, the SPA assesses interruptions during opening hours without reporting the actual opening hours of the facility. This is problematic, given that non-electrified facilities tend to have shorter opening hours [12]. Second, the inventory questionnaire asks about interruptions during the previous seven days of assessment and thereby ignores seasonal variations in the reliability of grid electricity, which occur in Malawi [24,25]. Third, the rationale for using a threshold of interruptions of no more than two hours at a time is unclear, as the relevance of interruptions for health service delivery is likely to depend not only on the duration of an interruption but also on the frequency and timing as well as the predictability of interruptions. Fourth, no information on the quality of the grid supply, the use of back-up sources or the functionality of solar systems is provided.

Regarding the functionality of energy uses, this primarily draws on self-reported data, which are verified through observation for a large proportion of the facilities. Apart from the energy uses *hematology analyzer*, *newborn incubator* and *computer*, the variables related to energy use (e.g. sanitation in outpatient area) are linked to, but not fully attributable, to the presence or absence of electricity. Although we aimed to take the demand of the facility into account by analyzing service-specific energy uses only in facilities where these services were offered, there might be further reasons that energy-specific uses are not required within the facility (e.g. newborn incubator required for advanced neonatal care services). It is unclear through which method the information regarding ventilation of the medication storage room was obtained.

Regarding the assessment of outputs and outcomes at facility level, most of the available variables are not suitable means for assessing the impacts of energy on health services. Our data analyses revealed that a major proportion of health facilities offering child delivery services lacks functional lighting in the respective area of the facility; this is particularly true for facilities offering night-time services. There was no statistically significant effect of the functionality of lighting in the delivery area on offering night-time services. This can probably be explained by the fact that our outcome variable assessed whether or not services are offered at all in a facility (which may not be affected by lighting) rather than assessing the quality of the services (which is likely to be affected by lighting). Likewise, our results indicate that although delivery and night-time care services are offered, functional lighting which would be indispensable for the quality of these services seems not to be prioritized in these settings. As lighting is less likely to be functional in off-grid settings, it will be important to explore specific barriers under these circumstances and to examine whether functional solar-powered systems are a suitable alternative for low-power, high-value appliances, in particular, lighting and refrigeration.

In general, assessing outputs or outcomes at facility level would require more in-depth assessment of a set of health facilities in relation to the services they intend to offer and the energy uses and underlying electricity and/or thermal energy needs required to facilitate delivery of these services. In this context, it will be important to take into account behavior change and adaptation mechanisms as a consequence of alterations in energy provision by those directly affected, in particular staff and patients. To track and understand more thoroughly the impact of changes over time, research designs other than cross-sectional analyses, for example intervention studies accompanied by in-depth qualitative examinations, are needed.

## Conclusion

The conceptual framework draws attention to the importance of energy in health facilities, highlighting that reliable electricity and thermal energy are required for the delivery of a broad range of health services. Recognizing the important role of energy is long overdue and should inform efforts to strengthen health systems in developing countries–at global, national and sub-national levels.

The results of the exploratory data analysis suggest that energy uses within facilities with a lower quality electricity supply tend to be less functional, independent of facility type, managing authority, region and urban-rural location. Consequently, these facilities are likely to be constrained in offering efficient and effective health services. In Malawi, electricity quality is particularly constrained in government-run, rural and smaller health facilities in the Central and Northern Regions, suggesting that these health facilities would particularly benefit from an improved energy supply.

The conceptual framework can help guide further research, including on the development of appropriate indicators which track access to and use of modern energy and its impact on health service delivery; these indicators should take complexity into account. In addition, the substantive gaps in the evidence base call for detailed energy audits in health facilities, qualitative studies examining the impacts of energy on health service delivery from the perspectives of multiple stakeholders as well as intervention studies addressing the shortcomings of electricity supply. This will further our as-of-yet incomplete understanding of the role of energy in health facilities, particularly by taking into account the behaviors and adaptations of staff and patients in relation to unreliable electricity. Ultimately, such research will help inform decision-makers about suitable strategies for enabling more effective and more equitable health service delivery through modern energy in health facilities.

## Supporting information

S1 Appendix (DOCX)

## Abbreviations

CHAM: Christian Health Association of Malawi
CICI: Context and Implementation of Complex Interventions
DHS: Demographic and Health Survey
GTF: Global Tracking Framework
HCWT: Healthcare Waste treatment
HAI: Health Action International
HIO: High Impact Opportunity
HVAC: Heating, Ventilation and Air Conditioning
ICT: Information and communication technologies
SDGs: Sustainable Development Goals
SE4ALL: Sustainable Energy For All
SPA: Service Provision Assessment
UN: United Nations
UNICEF: United Nations Children's Fund
USAID: United States Agency for International Development
WHO: World Health Organization
