# Supplementary material for: The role of energy in health facilities: A conceptual framework and complementary data assessment in Malawi
Source: PLoS One. 2018 Jul 20;13(7):e0200261. doi: 10.1371/journal.pone.0200261 (PMC6054392; doi:10.1371/journal.pone.0200261)
Supplement: S1 Appendix — (DOCX) [file pone.0200261.s001.docx]

S1 APPENDIX

Detailed description of conceptual framework

Energy types and characteristics of energy supply (usability)

Health facilities in sub-Saharan Africa may be supplied through a grid connection or through various off-grid solutions, both of which are most often supported through a back-up solution (1). Grid-electricity in sub-Saharan Africa is mainly derived from coal, oil, gas, hydro, wind and sometimes solar or geothermal sources (2). Additionally, combined heating power (CHP) systems are increasingly used in emerging economies (3, 4). Grid-connected health facilities usually use off-grid back-up solutions such as Photovoltaic (PV) systems with batteries, fuel-based generators or two-way battery generators to deal with power shortages which frequently occur in LMICs (3, 5).

Due to a lack of grid connection in rural areas in LMICs most of the health facilities in these regions rely on off-grid solutions (1, 6). In off-grid settings, commonly used energy types comprise hybrid systems (incl. PV system with batteries, wind turbines and/or a generator), diesel-generators, wind turbines and in some regions biogas plants for larger loads (3, 7, 8), and PV systems with batteries, gasoline- and propane-based generators and hydropower systems on small-scale level (9, 10). Back-up solutions in off-grid settings comprise mainly fuel-based generators (3).

Thermal energy generation comprises the direct combustion of solid (e.g. wood, coal, dung, etc.), liquid (diesel, kerosene, gasoline propane, etc.) and gaseous (e.g. natural gas, biogas, etc.) fuel (3, 11).

Regardless of the available energy type, there are three technical aspects directly affecting the potential to use energy devices within the health care setting. First, availability of the energy source plays a crucial role. Capacity relates to the quantity (measured in watts or in fuel) of energy available compared to energy needs; whereas timing and duration relate to the predictable time frame when energy is available in the health facility. The second component comprises reliability which is expressed as the timing and duration of energy availability without unpredictable shortages. Specifically, in the health care setting, non-reliable energy access may lead to significant resource loss in case of shortages. Third, if quality of electricity in terms of voltage and frequency (and caloric value, moisture and combustibility in the case of fuel combustion) is not ensured electronic devices may not function, or may even lead to increased risk of fire and injuries (9, 10, 12–15).

Apart from the three predominantly technical usability characteristics, the “soft” characteristic of *acceptability* has been shown to influence energy supply and maintenance at the household as well as at the community level (15–21). In this context, *acceptability* comprises the attitudes and, consequently, the behaviors concerning energy technologies. Socio-cultural perceptions (e.g. culturally anchored cook stoves fueled by solid biomass (16)), perceived convenience (e.g. workload for fuel procurement (18)), and perceived benefits or disadvantages (e.g. higher user satisfaction with PV power stations compared to small hydro power systems due to improved reliability (20)) may, inter alia, shape the attitudes and behaviors towards specific technologies. In the healthcare setting, acceptability might be reflected in the maintenance and efficient use of energy supply (10).

Energy uses and outputs at facility level

Adequate access to modern energy (assuming availability, quality, reliability and an energy-empowering context) enables the usage of energy in a variety of ways within the health facility. Every energy use type may in turn reinforce outputs at the facility level.

Energy allows for the lighting of indoor and outdoor spaces as well as specific lighting for clinical activities. Thus, lighting may ensure security on site, enable medical services (incl. emergency night-time care) and administrative tasks at night and promote safe clinical activities through improved visibility (9, 10, 12–15).

Through energy medical devices such as laboratory equipment and imaging methods for diagnostics^[[1]](#footnote-1)^ and surgical appliances for treatments^[[2]](#footnote-2)^ can be powered. Therefore, advanced diagnostics and treatments may be possible (3, 12, 13, 15, 22, 23).

Electricity is required to activate mechanized water pumps and therefore, enable access to improved water for drinking, medical activities, personal hygiene, cleaning and laundry, as well as to improved sanitation facilities. Additionally, electricity access allows the use of electric water treatment methods (e.g. ozone, reverse osmosis, UV disinfection and carbon filters) and onsite wastewater treatment. Functional sanitation- and sewerage-systems, as well as improved water access are crucial to create a safe facility environment. Safe water is needed to provide basic services and effectively prevent and treat diseases (e.g. water for child delivery, safe use of formula milk to prevent transmission of HIV from mother to child). Additionally, administrative efficiency may be empowered due to less time spent seeking personal needs (13, 15, 22–26).

Access to modern energy permits the utilization of information and communication technologies (ICT) in the form of phones (e.g. mobile- and radio-telephones), laptops, computers, technologies to obtain internet connection (V-Sat or cable) and more advanced e-Health technologies. Communication technologies may serve to request assistance from colleagues within a facility, as well as from other health facilities, for example, to obtain emergency medication or consultation, or to refer patients to these facilities. Other aims of ICT include surveillance and reporting of important factors, such as disease incidence and immunization. More advanced e-Health technologies comprise electronic medical records (EMR), clinical decision support systems (DSS) or training and education programs by means of computers, laptops, tablets or smartphones. ICT may improve health care infrastructure and logistics. Furthermore, (patient) data collection and monitoring by means of ICT may improve continuous treatment and serve to gather comprehensive data for research activities and decision-makers. Additionally, education and training may be enabled through e-learning opportunities (12, 13, 15, 24, 27–29).

Furthermore, modern energy access facilitates the use of refrigerators, which run type-dependent either with electricity (incl. standard- or ice lined compression) or with thermal energy (incl. absorption-, hybrid- and solar compression), and differ in their minimum holdover time (from 1.5 hours for absorption refrigerators to 20 hours for solar compression refrigerators). In the healthcare setting, refrigeration is required to cool not only food but also medical essentials such as blood, vaccines, medication, testing reagents and sera. These medical essentials lose their efficacy if continuous cooling is not ensured. Consequently, continuous cooling enables access to safe prevention, diagnostic and therapeutic resources. Additionally, food storage opportunities may improve nutrition, lower the risk of food acquired-infections (authors´ assumption) and generate time-savings in food preparations (authors´ assumption) (10, 13, 15, 23, 30–32).

Modern energy enables processing technologies for sterilization or disinfection of medical equipment for reuse, and of hazardous healthcare waste (HCW) such as sharps^[[3]](#footnote-3)^ and other non-chemically contaminated wastes (e.g. fluids, isolation and surgery waste and laboratory waste). Apart from sterilization and disinfection, HCW may be complemented by mechanical processes (e.g. shredder or grinder) to diminish the volume and make it unrecognizable. Non-electrical sterilization and disinfection solutions include stand-alone solutions (e.g. stoves, small-scale incinerators or solar-thermal autoclaves) meanwhile, in electrified settings, appliances such as autoclaves, microwaves, incinerators and other heat systems (e.g. dry heat systems) are used. Thus, on health facility level safe healthcare waste disposal (incl. sharps) and medical equipment use and reuse may be empowered (3, 33, 34).

Other thermal energy applications consist of Heating, Ventilation and Air Conditioning (HVAC) of spaces. On facility level, space cooling protects sensitive wards, operating theatres and laboratory equipment and therefore, guarantees their functionality (3, 10). Indoor temperature control may serve in extreme temperature health care settings to control infectious disease transmission and lower the health risk for both patients and staff (35–37).

Finally, energy access enables the usage of cooking facilities for food preparation and water boiling and other thermal water treatment appliances (e.g. distillation-, and pasteurization-technologies). Apart from safe drinking water, warm/hot water for medical activities (e.g. for child delivery) and safe food preparation opportunities may be facilitated (13, 15, 38).

Outcomes at facilities level

Across facilities, the outlined energy outputs may individually contribute to increased effectiveness and efficiency. Indicators of effectiveness include the timely utilization of needed health services and quality aspects in terms of efficacy (appropriate treatment), safety (minimization of infection risk) and continuity (e.g. continued access to care) (39). For example, the facilitated outputs of lighting might allow for the timely provision of needed health services during night-time, as well as foster efficacy and safety (12). Continuity might be enabled through e-Health technologies as DSS in the form of data collection and monitoring (29).

Efficiency encompasses costs and productivity as well as administrative efficiency which comprises the “value of health workers´ and patients´ time” (39). The cost implications of health-care-associated infections and inappropriate water and sanitation conditions in health care settings have not yet been determined but are expected to be substantial (25, 40, 41). Therefore, providing an energy infrastructure which tackles these safety concerns might be supportive to lower healthcare costs and improve productivity (in terms of effective treatment), too. Proxies for administrative efficiency comprise, inter alia, the availability of drugs and skilled personnel as well as health worker attrition and motivation (39). With regard to the availability of drugs, it has been found that reliable cooling was significantly stronger in electrified healthcare clinics compared to non-electrified facilities (30). As continuous cooling is crucial for specific medical essentials (e.g. vaccines), electricity access might be a key enabler to ensure the availability of effective drugs.

On the staff level, productivity might be ensured through time-savings. For example, with improved functional sanitation, health workers do not need to leave the facility to relieve themselves (12, 25). Additionally, improved cooking solutions as well as food refrigeration opportunities (authors´ assumption) might reduce the food preparation time due to time saved in fuel collection (18). Regarding health worker attrition and motivation, evidence suggests that health staff is generally restrained to live in rural areas without electricity access and health worker attendance has been observed to be significantly increased in electrified areas (13, 42). In a qualitative study conducted in Uganda it was depicted that physicians “eventually lose morale” and it is “very demotivating” for them if they aren´t able to operate because of missing medical devices. Furthermore, health staff expressed fear of contamination during night-time medical activities due to a lack of appropriate lighting which resulted in the perception of mistrust on the patients´ side (12). Consequently, energy access might improve health staff working conditions in terms of effectiveness and efficiency which in turn might allow these to offer more health services in a better quality.

Patients may therefore benefit from enabled effectiveness and efficiency by improved diagnostics and treatment which includes a better infection control and greater confidence and satisfaction (5, 12).

Impact on population level

On population level, energy access and use may facilitate access to care by fostering a facility infrastructure which allows facilities to supply a crucial range of health services (e.g. lighting appliances, refrigeration for vaccines). Evidence has been shown, that the care-seeking behavior of vulnerable groups is influenced by specific criteria of the health facility. For example, women may be deterred in seeking care to give birth in a health facility without improved water or sanitation facilities and the perception of quality of health services, in particular cleanliness and health workers´ kindness, is a decisive factor for women of seeking care (43, 44). This shows, that energy might be a critical enabler to provide the appropriate infrastructure to attract vulnerable population groups to seek care.

As described above, energy access and its use facilitates essential health services. Essential health services such as immunization services will be needed to tackle major disease burdens in developing countries (45–47). This implies that energy in health service delivery is required to reduce morbidity and mortality and improve the health status in the population.

Facility setting

Specific components of the health facility setting are strongly interconnected with energy access of a health facility. These include the facility type^[[4]](#footnote-4)^, building characteristics which refer to the infrastructure within the facility (e.g. energy efficiency aspects) as well as the availability of qualified workforce and facility management (9, 10).

Context

On the Macro level (national/regional context focusing on the layer *Energy types*), a country’s energy supply infrastructure influences energy access and use on health facility level (6). The political context in terms of governance and organizations as well as policies (e.g. policies of energy technology implementation or monitoring strategies) and standards may influence energy access in the health facility (6, 9, 13, 22, 50). The socio-economic atmosphere consists of available and accessible financial resources and funding mechanisms as well as regional and global energy markets (6, 22, 51). Furthermore, the legal context in terms of national laws and regulations may foster the deployment of efficient energy supply (9, 22, 50). The context on Meso level (facility context focusing on the layer *Energy uses*) comprises the political context in the form of programs (e.g. ICT strengthening programs) (52) and the epidemiological context. From an epidemiological perspective, developing and emerging economies do not only face a high burden of infectious diseases but are also confronted by the double burden of disease including chronic diseases which require specific equipment for detection and treatment (3, 53).

Environment and environmental health

The entire system, from energy type all the way to the impact at the population level may impact the immediate, as well as the global environment in several ways. Indoor-/ambient air-, water-, soil- and noise-pollution might be caused (e.g. air pollution through combustion of fuel, leak contamination due to poor energy management, noise exposure due to HCW technologies etc.) (3, 22, 33). Furthermore, waste disposal (e.g. nuclear-, post-combustion and electronic-waste) and climate change impacts (e.g. extreme weather events) remain a challenge of non-renewable energy types and energy uses (54, 55). Environmental health consequences might include waterborne-, airborne- and further climate-related diseases (e.g. vector-borne diseases), as well as fire and electrocution injuries/deaths and other injuries/deaths caused by climate change events (9, 55). Environmental aspects and resulting environmental health consequences might in turn feedback, affecting the other layers of the framework.

Bibliography: detailed description of conceptual framework

1. Adair-Rohani H, Zukor K, Bonjour S, Wilburn S, Kuesel AC, Hebert R et al. Limited electricity access in health facilities of sub-Saharan Africa: a systematic review of data on electricity access, sources, and reliability. Glob Health Sci Pract 2013 [cited 2017 Jan 9]; 1(2):249–61.

2. Marks J. African Energy Atlas; 2014 [cited 2017 Apr 23].

3. World Health Organization. Access to modern energy services for health facilities in resource-constrained settings: a review of status, significance, challenges and measurement; 2014.

4. Kerr T. Combined Heat and Power: Evaluating the Benefits of Greater Global Investement [cited 2017 Apr 27].

5. Winch P, Stepnitz R. Peak Oil and Health in Low- and Middle-Income Countries: Impacts and Potential Responses. Am J Public Health 2011; 101(9):1607–14.

6. Angelou N, Elizondo Azuela G, Portale E, Jaques Goldenberg I, Bhatia M, Banerjee SG et al. Global tracking framework; 2013 May 28. The World Bank 77889. Available from: URL: http://www-wds.worldbank.org/external/default/WDSContentServer/WDSP//.

7. Rupf GV, Bahri PA, Boer K de, McHenry MP. Broadening the potential of biogas in Sub-Saharan Africa: An assessment of feasible technologies and feedstocks. Renewable and Sustainable Energy Reviews 2016 [cited 2017 Apr 15]; 61:556–71.

8. Bensah EC, Brew-Hammond A. Biogas technology dissemination in Ghana: history, current status, future prospects, and policy significance. International Journal of Energy and Environment 1.2 2010 [cited 2017 Apr 15]:277–94.

9. United States Agency for International Development (USAID). Powering Health: Electrification Options for Rural Health Centers; 2009 [cited 2017 Jan 21]. Available from: URL: http://www.poweringhealth.org/Pubs/PNADJ557.pdf.

10. United States Agency for International Development (USAID). Powering Health: Energy Management in Health Facilities [cited 2017 Jan 21]. Available from: URL: http://www.poweringhealth.org/Pubs/powering_health_mgmt.pdf.

11. Orosz MS, Quoilin S, Hemond H. Technologies for heating, cooling and powering rural health facilities in sub-Saharan Africa. Proceedings of the Institution of Mechanical Engineers, Part A: Journal of Power and Energy 2013 [cited 2017 Mar 31]; 227(7):717–26.

12. Voluntary Service Overseas (VSO). Our Side of the Story - Ugandan health workers speak up [cited 2017 Jan 18]. Available from: URL: http://www.ivoindia.org/Images/VSO-Our-side-of-the-story_Ugandan-health-workers-speak-u_tcm78-35533.pdf.

13. Arvidson A, Songela F, Syngellakis K. The role of energy services in the health, education and water sectors and cross-sectoral linkages [cited 2017 Jan 18].

14. Brenneman A, Kerf M. Infrastructure & Poverty Linkages: A Literature Review; 2002. Available from: URL: http://ilo.org/wcmsp5/groups/public/---ed_emp/---emp_policy/---invest/documents/publication/wcms_asist_8281.pdf.

15. Jimenez A, Olson K. Renewable Energy for Rural Health Clinics; 1998 [cited 2017 Apr 6]. Available from: URL: http://www.nrel.gov/docs/legosti/fy98/25233.pdf.

16. Joon V, Chandra A, Bhattacharya M. Household energy consumption pattern and socio-cultural dimensions associated with it: A case study of rural Haryana, India. Biomass and Bioenergy 2009 [cited 2017 May 10]; 33(11):1509–12.

17. Müggenburg H, Tillmans A, Schweizer-Ries P, Raabe T, Adelmann P. Social acceptance of PicoPV systems as a means of rural electrification — A socio-technical case study in Ethiopia. Energy for Sustainable Development 2012 [cited 2017 May 10]; 16(1):90–7.

18. Troncoso K, Castillo A, Masera O, Merino L. Social perceptions about a technological innovation for fuelwood cooking: Case study in rural Mexico. Energy Policy 2007 [cited 2017 May 10]; 35(5):2799–810.

19. Barry M-L, Steyn H, Brent A. Selection of renewable energy technologies for Africa: Eight case studies in Rwanda, Tanzania and Malawi. Renewable Energy 2011 [cited 2017 May 10]; 36(11):2845–52.

20. Shyu C-W. End-users' experiences with electricity supply from stand-alone mini-grid solar PV power stations in rural areas of western China. Energy for Sustainable Development 2013 [cited 2017 May 10]; 17(4):391–400.

21. Loo JD, Hyseni L, Ouda R, Koske S, Nyagol R, Sadumah I et al. User Perspectives of Characteristics of Improved Cookstoves from a Field Evaluation in Western Kenya. Int J Environ Res Public Health 2016 [cited 2017 May 10]; 13(2):167.

22. Bhatia M, Angelou N. Beyond Connections: Energy access redefined: World Bank, Washington, DC; 2015. Available from: URL: https://openknowledge.worldbank.org/bitstream/10986/24368/1/Beyond0connect0d000technical0report.pdf.

23. Martinot E, Chaurey A, Lew D, Moreira JR, Wamukonya N. Renewable Energy Markets in Developing Countries. Annu. Rev. Energy. Environ. 2002 [cited 2017 Apr 16]; 27(1):309–48.

24. Cabraal RA, Barnes DF, Agarwal SG. PRODUCTIVE USES OF ENERGY FOR RURAL DEVELOPMENT. Annu. Rev. Environ. Resour. 2005 [cited 2017 Apr 5]; 30(1):117–44.

25. UNICEF, WHO. Water, sanitation and hygiene in health care facilities: status in low- and middle-income countries and way forward; 2015. Available from: URL: http://apps.who.int/iris/handle/10665/154588.

26. Chartier Y, Emmanuel J, Pieper U. Safe Management of Wastes from Health-care Activities: A Practical Guide. 2nd ed. Geneva: World Health Organization; 2014 [cited 2017 Apr 18].

27. Jason F. Cohen, Emma Coleman, and Lucienne Abrahams. Use and Impacts of E-health Within Community Health Facilities in Developing Countries: A Systematic Literature Review 2015 [cited 2017 Apr 16].

28. Wootton R, Patil NG, Scott RE. Telehealth in the Developing World; 2009.

29. Were MC, Shen C, Tierney WM, Mamlin JJ, Biondich PG, Li X et al. Evaluation of computer-generated reminders to improve CD4 laboratory monitoring in sub-Saharan Africa: a prospective comparative study. J Am Med Inform Assoc 2011 [cited 2017 Apr 26]; 18(2):150–5.

30. The World Bank. The Welfare Impact of Rural Electrification: A Reassessment of the Costs and Benefits Costs and Benefits: An IEG Impact Evaluation; 2008 [cited 2017 Jan 18]. Available from: URL: http://siteresources.worldbank.org/EXTRURELECT/Resources/full_doc.pdf.

31. Powering Health - Cold Chain and Refrigeration [cited 2017 Mar 29]. Available from: URL: http://www.poweringhealth.org/index.php/topics/technology/cold-chain-and-refrigeration#products.

32. Al-Akori A. PV Systems for Rural Health Facilities in Developing Areas: A completion of lessons learned; 2014 [cited 2017 Jan 25].

33. Emmanuel J. Compendium of Technologies for Treatment/Destruction of Healthcare Waste; 2012 [cited 2017 Jan 18].

34. Batterman S. Findings on an Assessment of Small-scale Incinerators for Health-care Waste; 2004 [cited 2017 May 1]. Available from: URL: http://www.who.int/water_sanitation_health/medicalwaste/en/smincinerators.pdf.

35. World Health Organization. Cooling and air conditioning [cited 2017 Mar 28]. Available from: URL: http://www.portal.pmnch.org/sustainable-development/housing/strategies/air-conditioning/en/.

36. Wright CY, Street RA, Cele N, Kunene Z, Balakrishna Y, Albers PN et al. Indoor Temperatures in Patient Waiting Rooms in Eight Rural Primary Health Care Centers in Northern South Africa and the Related Potential Risks to Human Health and Wellbeing. Int J Environ Res Public Health 2017; 14(1).

37. Hersoug L‐G. Viruses as the causative agent related to ‘dampness’ and the missing link between allergen exposure and onset of allergic disease. Indoor Air 2005; 15(5):363–6. Available from: URL: http://onlinelibrary.wiley.com/doi/10.1111/j.1600-0668.2005.00382.x/full.

38. Lantagne D. Household Water Treatment Options in Developing Countries: Boiling [cited 2017 Apr 16].

39. Kruk ME, Freedman LP. Assessing health system performance in developing countries: a review of the literature. Health Policy 2008 [cited 2017 Apr 25]; 85(3):263–76.

40. Strohmeier H. Why sustainable energy matters to children: The critical importance of sustainable energy for children and guture generations; 2015 [cited 2017 Jan 25].

41. Allegranzi B, Nejad SB, Combescure C, Graafmans W, Attar H, Donaldson L et al. Burden of endemic health-care-associated infection in developing countries: Systematic review and meta-analysis. The Lancet 2011 [cited 2017 May 12]; 377(9761):228–41.

42. Chaudhury N. Ghost Doctors: Absenteeism in Rural Bangladeshi Health Facilities. The World Bank economic review 2004; 18(3):423–41.

43. Velleman Y, Mason E, Graham W, Benova L, Chopra M, Campbell OMR et al. From Joint Thinking to Joint Action: A Call to Action on Improving Water, Sanitation, and Hygiene for Maternal and Newborn Health. PLOS Medicine 2014; 11(12):e1001771. Available from: URL: http://journals.plos.org/plosmedicine/article/file?id=10.1371/journal.pmed.1001771&type=printable.

44. Pembe AB, Urassa DP, Darj E, Carlsted A, Olsson P. Qualitative study on maternal referrals in rural Tanzania: decision making and acceptance of referral advice. Afr J Reprod Health 2008; 12(2):120–31.

45. Brenzel L, Wolfson LJ, Fox-Rushby J, Miller M, Halsey NA. Vaccine-preventable Diseases: The International Bank for Reconstruction and Development / The World Bank; 2006.

46. World Health Organization. The top 10 causes of death; 2017 [cited 2017 May 18]. Available from: URL: http://www.who.int/mediacentre/factsheets/fs310/en/index1.html.

47. World Health Organization. WHO Model List of Essential Medicines 14th edition (March 2005) [cited 2017 Mar 29].

48. World Health Organization. Baseline country survey on medical devices; 2010 [cited 2017 May 18].

49. Jamison DT, Breman JG, Measham AR, Alleyne G, Claeson M, Evans DB et al. Providing Interventions: The International Bank for Reconstruction and Development / The World Bank; 2006.

50. Steuwer DS. Energy Efficiency Governance. Wiesbaden: Springer Fachmedien Wiesbaden; 2013 [cited 2017 Apr 17].

51. Practical Action. Poor people´s energy outlook 2016: National Energy Access Planning from the Bottom Up; 2016 [cited 2017 Feb 15].

52. Nyamtema A, Mwakatundu N, Dominico S, Kasanga M, Jamadini F, Maokola K et al. Introducing eHealth strategies to enhance maternal and perinatal health care in rural Tanzania. Maternal Health, Neonatology and Perinatology 2017; 3(1):3. Available from: URL: https://mhnpjournal.biomedcentral.com/track/pdf/10.1186/s40748-017-0042-4?site=mhnpjournal.biomedcentral.com.

53. Aikins Ad-G, Unwin N, Agyemang C, Allotey P, Campbell C, Arhinful D. Tackling Africa's chronic disease burden: from the local to the global. Global Health 2010; 6(1):5. Available from: URL: http://globalizationandhealth.biomedcentral.com/track/pdf/10.1186/1744-8603-6-5?site=globalizationandhealth.biomedcentral.com.

54. Built Environment — The Collaborative on Health and the Environment [cited 2017 May 19]. Available from: URL: https://www.healthandenvironment.org/what-we-know/environmental-contributors/built-environment.

55. Prüss-Üstün A, Wolf J, Corvalán C, Bos R, Neira M. Preventing disease through healthy environments: A global assessment of the burden of disease from environmental risks. Second edition. Geneva Switzerland: World Health Organization; 2016 [cited 2017 May 19].

Sensitivity analysis

**Table 1A. Lighting availability in the delivery area in facilities offering night-time care and delivery services (in %)**

|  |  | *N* | *C1* | *C2* | *C3* | *C4* | *C5* | *C6* |
| --- | --- | --- | --- | --- | --- | --- | --- | --- |
| **Inpatient** | *Lighting available* | 18 | 70.52 | 73.89 | 47.71 | 55.41 | 40.55 | 9.98 |
| **services** | *Lighting not available* | 32 | 29.48 | 26.11 | 52.29 | 44.59 | 59.45 | 90.02 |
| **Overnight** | *Lighting available* | 93 | 70.52 | 73.89 | 47.71 | 55.41 | 40.55 | 9.98 |
| **observation** | *Lighting not available* | 164 | 29.48 | 26.11 | 52.29 | 44.59 | 59.45 | 90.02 |
| **No inpatient/** | *Lighting available* | 115 | 100.00 | 49.28 | 16.33 | 47.24 | 29.15 | 0.00 |
| **Overnight** | *Lighting not available* | 87 | 0.00 | 50.72 | 83.67 | 52.76 | 70.85 | 100.00 |

C1: Uninterrupted grid with back-up; C2: Interrupted grid with back-up; C3: Uninterrupted grid without back-up; C4: Interrupted grid without back-up; C5: Off-grid electricity source; C6: No electricity source

**Table 2A. Sensitivity analysis: Lighting in outpatient areas, Lighting in delivery area, Computer, Communication technologies, Technologies for sterilization and disinfection, Light microscope, Hematology analyzer, Newborn incubator: coefficient and standard error and odds ratios of energy source and continuity**

|  | Lighting in outpatient area | | | Lighting in delivery area | | | Computer | | Communication technologies | | |
| --- | --- | --- | --- | --- | --- | --- | --- | --- | --- | --- | --- |
|  |  | |  |  | |  |  |  |  | |  |
|  | Available (=1) vs. Not available | | | Available (=1) vs. Not available | | | Available (=1) vs. Not available | | Available (=1) vs. Not available | | |
|  |  |  | |  |  | |  |  |  |  | |
|  | β (SE β) | OR | | β (SE β) | OR | | β (SE β) | OR | β (SE β) | OR | |
| Constant | 0.59 (0.44) | 1.80 | | 1.04 (0.93) | 2.84 | | 1.74^***^ (0.55) | 5.68 | 1.53^**^ (0.61) | 4.62 | |
| C1 | ref |  | | ref |  | | ref |  | ref |  | |
| C2 | -0.28 (0.35) | 0.76 | | 0.17 (0.49) | 1.18 | | -0.74 (0.45) | 0.48 | 0.36 (0.59) | 1.44 | |
| C3 | -1.00^***^ (0.36) | 0,37 | | -0.98^*^ (0.52) | 0.38 | | -2.08^***^ (0.44) | 0.13 | 0.62 (0.59) | 1.86 | |
| C4 | -1.16^***^ (0.34) | 0.31 | | -0.65 (0.49) | 0.52 | | -2.48^***^ (0.43) | 0:08 | 0.20 (0.53) | 1.22 | |
| C5 | -1.53^***^ (0.37) | 0.22 | | -1.39^***^ (0.50) | 0.25 | | -4.30^***^ (0.52) | 0.01 | -0.42 (0.56) | 0.66 | |
| C6 | -2.58^***^ (0.48) | 0.08 | | -2.31^***^ (0.68) | 0.19 | | -5.52^***^ (1.11) | 0.00 | -0.78 (0.60) | 0.46 | |
| n | 950 |  | | 536 |  | | 972 |  | 972 |  | |
| Likelihood ratio test χ² | 175.98^***^ |  | | 77.98^***^ |  | | 486.63^***^ |  | 61.74^***^ |  | |
| Nagelkerke pseudo R² | 0.23 |  | | 0.18 |  | | 0.55 |  | 0.13 |  | |

C1: Uninterrupted grid with back-up; C2: Interrupted grid with back-up; C3: Uninterrupted grid without back-up; C4: Interrupted grid without back-up; C5: Off-grid electricity source; C6: No electricity source; OR: Odds ratio; SE: Standard Error; LR: Likelihood ratio; *** p<0.01, ** p<0.05; All regressions controlled for: Facility level, managing authority, region, urban/rural location

**Table 2A. continued**

|  | Sterilization and disinfection technologies | | Light microscope | | Hematology analyzer | | Newborn incubator | |
| --- | --- | --- | --- | --- | --- | --- | --- | --- |
|  |  |  |  |  |  |  |  |  |
|  | Available (=1) vs. Not available | | Available (=1) vs. Not available | | Available (=1) vs. Not available | | Available (=1) vs. Not available | |
|  |  |  |  |  |  |  |  |  |
|  | β (SE β) | OR | β (SE β) | OR | β (SE β) | OR | β (SE β) | OR |
| Constant | 1.73^***^ (0.66) | 5.64 | 0.10 (0.52) | 1.09 | 0.15 (0.89) | 1.16 | -2.25^*^ (1.26) | 0.11 |
| C1 | ref |  | ref |  | ref |  | ref |  |
| C2 | -0.33 (0.58) | 0.72 | -0.38 (0.37) | 0.69 | -0.30 (0.44) | 0.74 | -0.02 (0.48) | 0.98 |
| C3 | -1.17^**^ (0.55) | 0.31 | -1.67^***^ (0.40) | 0.19 | -1.98^***^ (0.60) | 0.14 | -2.18^***^ (0.73) | 0.11 |
| C4 | -1.44^***^ (0.53) | 0.24 | -1.79^***^ (0.37) | 0.17 | -3.10^***^ (0.65) | 0.05 | -2.10^***^ (0.60) | 0.12 |
| C5 | -3.85^***^ (0.58) | 0.02 | -3.08^***^ (0.47) | 0.05 | -18.56 (1,343.61) | 0.00 | -3.26^***^ (0.78) | 0.04 |
| C6 | -4.31^***^ (0.80) | 0.01 | -2.86^***^ (0.63) | 0.06 | -18.38 (2,435.83) | 0.00 | -17.66 (1,153.06) | 0.00 |
| n | 793 |  | 800 |  | 267 |  | 536 |  |
| LR test χ² | 382.38^***^ |  | 199.76^***^ |  | 129.59^***^ |  | 137.99^***^ |  |
| Nagelkerke pseudo R² | 0.51 |  | 0.32 |  | 0.44 |  | 0.44 |  |

C1: Uninterrupted grid with back-up; C2: Interrupted grid with back-up; C3: Uninterrupted grid without back-up; C4: Interrupted grid without back-up; C5: Off-grid electricity source; C6: No electricity source; OR: Odds ratio; SE: Standard Error; LR: Likelihood ratio; *** p<0.01, ** p<0.05; All regressions controlled for: Facility level, managing authority, region, urban/rural location

1. X-ray machines, microscopes, centrifuges, mixers, ultrasounds etc. (sources in text) [↑](#footnote-ref-1)
2. Suction machines, incubators, nebulizers, oxygen concentrator, pulse oximeters, heart monitors, anesthesia machines, defibrillators, electrocardiographs etc. (sources in text) [↑](#footnote-ref-2)
3. Sharps may consist of hypodermic, intravenous or other needles; autodisable syringes; syringes with attached needles; infusion sets; scalpels; pipettes; knives; blades; broken glass (33)(38) [↑](#footnote-ref-3)
4. The presented classification of facility types is based on the classification by the World Health Organization (WHO) (48)(53) and World bank (49)(54) [↑](#footnote-ref-4)
